# Respiratory pattern and phrenic and hypoglossal nerve activity during normoxia and hypoxia in 6-OHDA-induced bilateral model of Parkinson’s disease

**DOI:** 10.1186/s12576-020-00743-4

**Published:** 2020-03-11

**Authors:** Kryspin Andrzejewski, Monika Jampolska, Małgorzata Zaremba, Ilona Joniec-Maciejak, Paweł M. Boguszewski, Katarzyna Kaczyńska

**Affiliations:** 1 Department of Respiration Physiology, Mossakowski Medical Research Centre, Polish Academy of Sciences, Pawińskiego 5, 02-106 Warsaw, Poland; 2 Department of Experimental and Clinical Pharmacology, Centre for Preclinical Research (CePT), Medical University of Warsaw, Warsaw, Poland; 3 Laboratory of Animal Models, Neurobiology Centre, Nencki Institute of Experimental Biology of Polish Academy of Sciences, Warsaw, Poland

**Keywords:** Parkinson’s disease, 6-OHDA rat model, Hypoxic ventilatory response, Dopaminergic receptor, Phrenic and hypoglossal nerves

## Abstract

Respiratory disturbances present in Parkinson’s disease (PD) are not well understood. Thus, studies in animal models aimed to link brain dopamine (DA) deficits with respiratory impairment are needed. Adult Wistar rats were lesioned with injection of 6-hydroxydopamine (6-OHDA) into the third cerebral ventricle. Two weeks after hypoxic test was performed in whole-body plethysmography chamber, phrenic (PHR) and hypoglossal (HG) nerve activities were recorded in normoxic and hypoxic conditions in anesthetized, vagotomized, paralyzed and mechanically ventilated rats. The effects of activation and blockade of dopaminergic carotid body receptors were investigated during normoxia in anesthetized spontaneously breathing rats. 6-OHDA injection affected resting respiratory pattern in awake animals: an increase in tidal volume and a decrease in respiratory rate had no effect on minute ventilation. Hypoxia magnified the amplitude and minute activity of the PHR and HG nerve of 6-OHDA rats. The ratio of pre-inspiratory to inspiratory HG burst amplitude was reduced in normoxic breathing. Yet, the ratio of pre-inspiratory time to total time of the respiratory cycle was increased during normoxia. 6-OHDA lesion had no impact on DA and domperidone effects on the respiratory pattern, which indicate that peripheral DA receptors are not affected in this model. Analysis of monoamines confirmed substantial striatal depletion of dopamine, serotonin and noradrenaline (NA) and reduction of NA content in the brainstem. In bilateral 6-OHDA model changes in activity of both nerves: HG (linked with increased apnea episodes) and PHR are present. Demonstrated respiratory effects could be related to specific depletion of DA and NA.

## Introduction

Parkinson’s disease (PD) is a long-term neurodegenerative disorder of the central nervous system (CNS). Patients with PD apart from deficits in motor activity exhibit respiratory system impairments such as: dyspnea, sleep breathing disorders, restrictive pulmonary function, and upper airway dysfunction [1, 2]. Scarce respiratory studies in PD patients have not explained the origin of ventilatory abnormalities appearing in the disease [3–6]. Therefore, studies in animal models of PD, aimed to link brain DA deficits with respiratory disturbances are necessary to investigate mechanisms involved. They allow the researcher to obtain more homogenous stage of PD pathology and to eliminate the impact of antiparkinsonian drug treatment in modeled animals. To date 6-hydroxydopamine (6-OHDA) [7, 8] and reserpine models have been used [9] for studying respiratory impairments present in PD.

Recent study performed in 6-OHDA-induced PD model demonstrated decrease in expression of neurokinin 1 receptor in the pre-Bötzinger Complex and phox2b-expressing neurons in retrotrapezoid nucleus corresponding with frequency of breathing decline in normoxic breathing and decreased tachypneic response to hypercapnia [8, 10]. Our previous study in unilateral 6-OHDA model displayed increased tidal volume and attenuated respiratory rate responses during hypoxia and hypercapnia in comparison to sham-operated rats [11, 12].

DA and dopaminergic receptors have been indicated to play an important role in the regulation of breathing [13, 14]. Dopaminergic axons are distributed in the carotid bodies and within the brainstem where respiratory neurons are located [14]. It has been indicated that dopamine suppresses responsiveness to hypoxia, both in the central chemoreflex pathway and in the carotid body [15–18]. Therefore, it seems that dopamine neurons degeneration, DA deficiency and altered expression of dopaminergic receptors may lead to disturbed breathing control.

In the present study, we investigated normoxic pattern of breathing and hypoxic respiratory response in awake rats in the bilateral model evoked with intracerebroventricular (ICV) 6-OHDA injection, imitating advanced stage of PD [19]. Further we examined the activity of hypoglossal (HG) and phrenic (PHR) nerves after exposure to acute hypoxia, for the first time in bilateral PD model, having in mind that in unilateral one contralateral intact hemisphere may have compensatory influence on respiratory reflexes.

HG nerve innervates tongue muscles and its pre-inspiratory activity secures patency of the pharyngeal airway preparing upper airway muscles for inspiration [7]. Dysfunction of upper airway is one of the respiratory disturbances observed in PD patients [1], therefore analysis of HG nerve activity (pre-inspiratory time and amplitude) in PD model seems to be valuable tool for studying foregoing.

We have investigated previously the respiratory response to acute hypoxia after blockade of D_2_ receptors in 6-OHDA unilateral model [11]. All available studies in animal models of PD focus on the central effects of DA depletion. Potential changes in the peripheral dopaminergic receptors are neglected. Therefore, last purpose of the current study was examination of an impact of central DA depletion on stimulation and blockade of peripheral carotid body dopaminergic receptors in 6-OHDA-lesioned rats. Both tested agents dopamine and domperidone act only peripherally after systemic application [20, 21]. We decided to test this only during air breathing because in the current study ICV 6-OHDA lesion affected only normoxic pattern of breathing.

Behavioral test and contents of DA, noradrenaline (NA) and serotonin (5-HT) were analyzed in the striatum and brainstem to confirm lesion effectiveness and to allow interpretation of the respiratory test results. To avoid any compensation the study was performed 2 weeks after lesion. The rats were also pretreated with desipramine to protect noradrenergic neurons and obtain more selective degeneration of the dopaminergic system [19].

## Material and methods

### Animals and experimental protocol

All experimental procedures were approved by the local Ethics Committee for Animal Experimentation and conformed with the international/EU guidelines and regulations on the use and care of laboratory animals (EU Directive 2010/63/EU for animal experiments). Young adult male Wistar rats (*n* = 29) weighing 230–260 g (10–12 weeks old) at the beginning of the experiment were housed under standard laboratory conditions with a 12 h light/12 h dark cycle and unrestricted access to food and water.

The experiments were performed in the following scheme:

I. Hypoxic tests in awake rats in plethysmographic chamber and open field test before and 14 days after 6-OHDA/vehicle ICV administration: Rats from plethysmographic chamber experiments were re-used the following day:
II. Hypoxic test in anesthetized animals with registration of phrenic and hypoglossal nerve activity 15 days after 6-OHDA/vehicle ICV administration.
  1. Sham rats injected ICV with vehicle (*n* = 7).
  2. ICV 6-OHDA-injected rats (*n* = 9).
III. Effects of D_2_ receptor agonist (dopamine) and antagonist (domperidone) (iv) injection, on normoxic ventilatory parameters in anesthetized rats 14 days after 6-OHDA/vehicle ICV administration.
  1. Sham rats injected ICV with vehicle (*n* = 6).
  2. ICV 6-OHDA-injected rats (*n* = 7).

### 6-OHDA bilateral model

Rats were anesthetized with an intraperitoneal injection of thiopentalum natricum (Sandoz GmbH, Austria) at a dose of 90 mg kg^−1^ and fixed to a stereotaxic frame (Digital Lab Standard Stereotaxic Stoelting, USA). Desipramine hydrochloride (25 mg kg^−1^, Sigma Aldrich, Poland) was given ip 30 min before the surgery to preclude the uptake of 6-OHDA by noradrenergic nerve terminals. Operation was performed using standard aseptic technique. After skin incision, the skull was trephined with a dental drill in specific stereotaxic coordinates according of the Paxinos and Watson Atlas [22]. Stereotaxic coordinates for the administration site were antero-posterior, bregma: − 0.85 mm, lateral: 0.0 mm, ventral dura: − 7.1 mm. Vehicle or 6-hydroxydopamine hydrochloride (250 μg dissolved in 0.9% NaCl containing 0.1% ascorbic acid—Sigma Aldrich, Poland) at a volume of 10 μl was injected into the third brain ventricle with a sterile Hamilton micro-syringe with rate of 1 μl min^−1^. The dose of 6-OHDA applied in the present study was selected based on the literature [23, 24]. After injection, the needle was left in the brain for 5 min to prevent the solution from flowing backward and then was slowly withdrawn. After the surgery, rats were left to recover with standard laboratory conditions, and unlimited access to food and water. One day before lesion and 2 weeks later behavioral and hypoxic tests were carried out.

### Measurement of lung ventilation in awake rats

Ventilation and its response to acute hypoxia were investigated in a whole-body rodent plethysmography (WBP, model PLY 3223, Buxco Electronics, USA), as described previously [10]. Briefly, the calibration of volume was performed during each experiment by injecting of 1 ml of air into the chamber. The pressure signal was amplified, filtered, recorded, and analyzed with data analysis software (Biosystem XA for Windows, SFT3410 230 ver 2.9; Buxco Electronics, Wilmington, NC) generating tidal volume (V_T_, ml) and breathing frequency (f, breaths min^−1^). Tidal volume was calculated using approach of Epstein et al. [25]. Minute ventilation (V_E_, ml min^−1^, BTPS) was determined as a product of tidal volume and breathing frequency. V_T_ and V_E_ were normalized to body weight (ml kg^−1^ and ml kg^−1^ min^−1^, respectively). Rectal temperature was measured before and at the end of the experiments, and the values were averaged. All experiments were performed at room temperature (24–26 °C). Each rat was placed in the chamber (4.7 L) and left for 30 min of adaptation, while flushing with fraction of atmospheric air at 2.5 L min^−1^ to prevent CO_2_ accumulation. Acute hypoxia was achieved by a rapid flushing of gas mixture containing 8% of O_2_ in N_2_. Ventilation and its response to inspired hypoxia before and after implementation of PD model were registered. After 30 min of adaptation to breathing with the chamber air, pulmonary ventilation was taken as the baseline level of ventilation and recorded during 1 min before the introduction of hypoxia. Ventilation during 3 min of hypoxia and 5 min after switching to the air breathing was recorded. Period of 30 s breathing preceding hypoxia was calculated as a control normoxic breathing. Three-minute period of hypoxia breathing was averaged.

### Electrophysiological experiments in anesthetized rats

Two weeks after 6-OHDA or vehicle ICV administration rats were anesthetized intraperitoneally with 750 mg/kg of urethane (Sigma Aldrich, Poland) and 150 mg/kg of α-chloralose (Fluka, Germany). Animals were cannulated into the femoral artery to monitor blood pressure and femoral vein to administer supplemental anesthesia and fluids as required. Rectal temperature was maintained throughout the experiment at 37–38 °C via external heating pad. Arterial blood pressure was measured with a BP-2 Columbus Instruments (Columbus, USA). After the tracheostomy and pipecuronium bromide (Arduan, Gedeon-Richter, Hungary) administration at initial dose of 0.08 mg/kg (supplemented every hour) paralyzed rats were artificially ventilated (7025 Rodent Ventilator, Ugo Basile, Comerio, Italy). End-tidal CO_2_ was maintained between 4.5 and 5.0% (Capstar-100, CWE, USA) by adjusting parameters of mechanical ventilation (tidal volume was 2.3 ml, respiratory rate ranged between 35–40 strokes/min). The exhalation port of the ventilator was attached to a positive pressure of 3 cm H_2_O to prevent alveolar collapse. Vagal nerves were isolated in the neck and cut to eliminate an adjustment of the respiratory activity with lung inflation caused by respiratory pump. The phrenic nerve root and the hypoglossal nerve were transected in the neck. Central ends of the whole phrenic nerve and the main hypoglossal trunk were placed on bipolar silver electrodes for recording. The activities of both nerves were amplified and filtered (5–2500 Hz) using a NeuroLog system (Digitimer Ltd., Welwyn, UK) and integrated with the time constant of 70 ms. Raw and integrated nervous activities and arterial blood pressure were digitized using a CED Power 1401 data acquisition interface, recorded on a computer and analyzed using the Spike 2 software (Cambridge Electronic Design, Cambridge, UK). Acute hypoxia protocol consisted of ventilation with 8% oxygen in nitrogen. Each hypoxic exposure lasted 1.5 min or was stopped when episode of apnea occurred.

All analyzed data were calculated from the integrated phrenic and hypoglossal respiratory neurograms, as displayed previously [26]. Frequency (f), inspiratory time (Ti), expiratory time (Te), and the respiratory cycle time (Tc) were computed based on the activity of the phrenic nerve. The beginning of Ti was set as the start of the increase in the integrated activity of the PHR. The end of Ti was determined at a point where the maximal amplitude of the PHR decreased by 50%. Te was calculated as a period between the end and the beginning of Ti. Tc was a sum of Ti and Te. The amplitude of the pre-inspiratory HG activity (A pre-I HG) was determined based on the integrated hypoglossal activity at the time point corresponding to the start of the phrenic nerve activity. The time between the onset of the integrated HG activity and that of the PHR activity indicated the duration of the pre-inspiratory hypoglossal activity (T pre-I HG). All parameters were recorded and determined by averaging the variables measured for 15 s at the air-breathing, during each hypoxic episode (averaged periods of 15–30 s, 30–45 s, 45–90 s) and at recovery (1 min after the end of hypoxia). Changes in amplitude, frequency and minute activity were expressed as a percentage of the baseline of nerve activity and reported as means ± SEM. The duration of the T pre-I HG was calculated relative to the duration of the respiratory cycle measured between the onsets of integrated hypoglossal bursts (T pre-I/Tc), whereas the amplitude of the pre-I HG was presented as a fraction of the peak inspiratory hypoglossal amplitude (A pre-I/A HG).

#### Respiratory response after dopamine and domperidone injection in spontaneously breathing anesthetized rats

14 days after 6-OHDA lesion rats were anesthetized and prepared in similar way to procedure described in previous section excluding vagi nerve dissection, nerves preparation and arduan application. The tracheal cannula was connected to a pneumotachograph head, linked to Research Pneumotach System (RSS 100 h, Hans Rudolph Inc.) and a computerized recording system (Windows software version 3.07.02, KORR Medical Technologies Inc.) for measuring and recording tracheal airflow, respiratory frequency (f), tidal volume (V_T_), respiratory minute volume (V_E_).

Dopamine (Sigma Aldrich Poland) at a dose of 20 µg/kg and subsequently domperidone (Sigma Aldrich Poland) at a dose of 1 mg/kg, both dissolved in 0.9% saline (Sigma Aldrich Poland) were administered into the femoral vein in a volume of 0.2 ml. After injection catheter was immediately flushed with 0.2 ml of physiological saline. The gap between drug injections was 10 min.

The ventilatory parameters like V_T_ and V_E_ were calculated to body mass and by averaging the variables measured for 15, 30, 60 s, 2 min, 3 min and 5 min after the challenge just prior to drug injection and at selected time points of maximal respiratory change. Maximum of the response chosen from measured time points and recovery phase were presented.

### Behavioral open field test

To test whether 6-OHDA/vehicle ICV injected rats present any behavioral changes the animals were examined in the open field before and 14 days after administration. The test gives possibility of estimation of animal locomotor activity, exploration and motivation. The open field test was performed in a plastic chamber (size 75 × 75 × 35 cm) in infrared light without daylight. Each animal was put on the central place of the open field and was registered during 15 min by video camera. Results were analyzed by associated software (EthoVision XT and BehaActive) to count the parameters describing the vertical movement and exploration.

#### High-performance liquid chromatography (HPLC) analysis: assay of dopamine, serotonin, and noradrenaline and parallel metabolites

The animals were killed by decapitation 14 days following 6-OHDA or sham operation to III ventricle, then the brains were rapidly removed. The left/right striata and brainstem were dissected. Each tissue sample was weighed, placed on dry ice and frozen (− 80 °C) until further biochemical analysis. Thereafter, the brain tissue samples were homogenized with the ice-cold 0.1 M HClO_4_ containing 0.05 mM ascorbic acid and centrifuged at 13,000×*g* for 15 min at 4 °C. The supernatant was filtered through 0.2-μm pore size filter (Whatman, UK). The high-performance liquid chromatography system with electrochemical detection (HPLC-ED) comprising an electrochemical detector (L-3500 detector; Merck, Germany) with a glassy carbon electrode, autosampler injector (Merck) and delivery pump (Knauer, Germany) was used to measure the content of either DA, 5-HT and NA, together with parallel metabolites: 3,4-dihydroxyphenylacetic acid (DOPAC), homovanillic acid (HVA), 5-hydroxyindolacetic acid (5-HIAA), 3-methoxy-4-hydroxyphenylglycol acid (MHPG). The voltage was setting at + 0.8 V with respect to an Ag/Ag Cl reference electrode. The aliquots (20 μl) were separated on the C–18 a reverse phase column (250 × 4.6 mm; Nucleosil, 5 μm, Macherey–Nagel, Germany). A mobile phase containing: 32 mM sodium phosphate (Sigma-Aldrich, USA), 34 mM citric acid (Sigma-Aldrich, USA), 1 mM octane sulfonic acid (Sigma-Aldrich, USA), and 54 μM ethylenediaminetetraacetic acid (EDTA; Sigma-Aldrich, USA) in deionized (18.3 mΩ) water with 16% methanol (Merck, Germany). The mobile phase was infused at a flow rate of 0.8 ml min^−1^_._ Quantitation was achieved by comparing the peak area ratios of analyte to the external standard calibration curve with ClarityChrom software (Knauer, Germany). The contents of monoamines and parallel metabolites were expressed as pg mg^−1^ fresh tissue.

### Statistical analysis

Data were analyzed using non-parametric statistics. Differences between sham and 6-OHDA groups were evaluated with Whitney–Mann U test. For comparison within the group, Wilcoxon’s signed-ranks test was used. The confidence limit of *p* < 0.05 was considered to be statistically significant. The results are expressed as the means ± SEM.

## Results

### Hypoxic ventilatory response in vehicle and 6-OHDA-injected awake rats

**Fig. 1:**
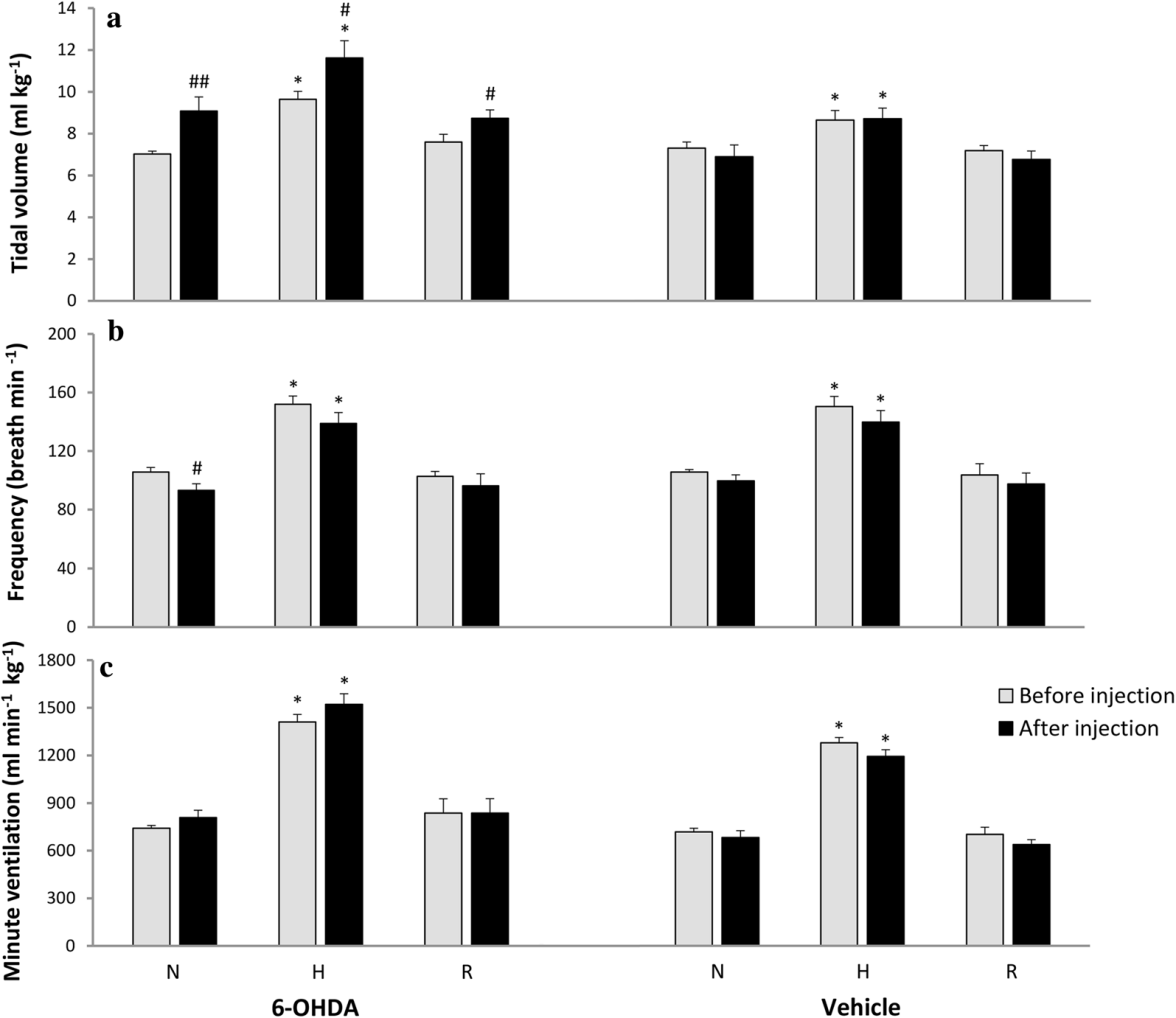
Respiratory response to hypoxic stimulus before (grey bar) and 14 days (black bar) after ICV injection of vehicle (sham) or 6-OHDA. **a** Tidal volume; **b** frequency of breathing; **c** minute ventilation. *N *normoxia, *H *hypoxia, *R *recovery after the hypoxic stimulus. All values are mean ± SEM. *^#^*p* < 0.05; ^##^*p* < 0.01. *—statistical significance versus the respective normoxic baseline value (N), #—statistical significance versus the corresponding pre-injection with 6-OHDA value (*n* = 7–9 per group)

Vehicle injection into the third brain ventricle had no influence on normoxic and hypoxic values of all respiratory parameters in sham rats (Fig. 1). 6-OHDA administration produced a statistically significant 23% increase in tidal volume and 12% decrease in frequency of breathing during normoxia (Fig. 1a, b). In comparison to pre-6-OHDA state respiratory response of V_T_ showed significant increase during hypoxia (18%), and at recovery (13%), while respiratory rate during hypoxia showed nonsignificant decline (9%). The alteration between pre-lesion and after 6-OHDA injection in hypoxic respiratory response seems to be the consequence of changes in resting respiratory variables, however in PD model rats V_T_ response during hypoxia seems to be attenuated: it increased above 28% of its normoxic value, while before 6-OHDA injection V_T_ was able to rise above 37% of its control. Changes in V_T_ and f were inverse and produced nonsignificant increase of V_E_ in normoxic (8%) and low oxygen condition (7%) in comparison to pre-lesion state (Fig. 1c).

#### Phrenic nerve activity under normoxic and hypoxic condition in vehicle and 6-OHDA-treated rats

**Fig. 2:**
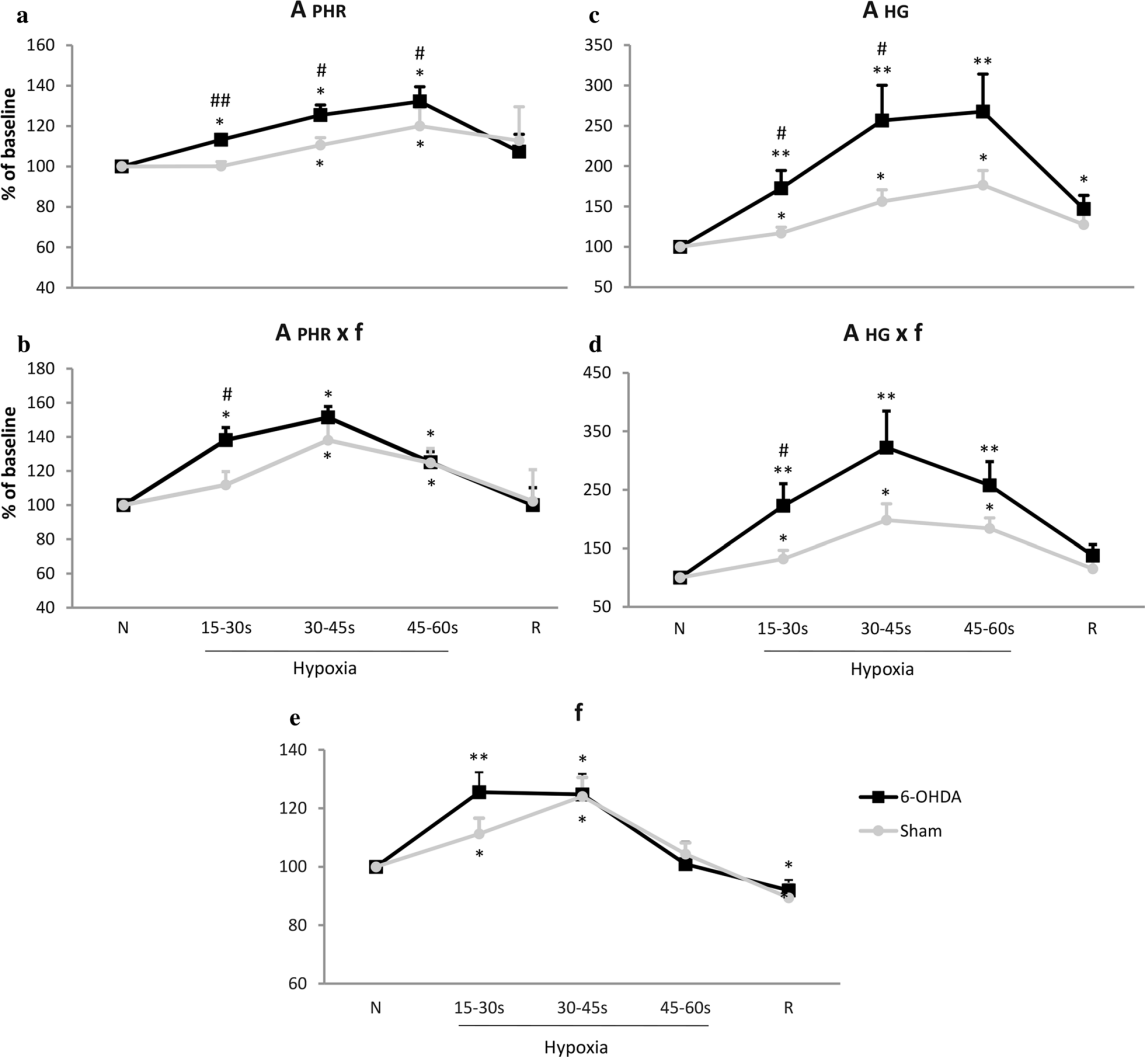
Changes of amplitude (A), minute activity (A × f) and frequency of discharge (f) of the phrenic nerve (PHR) (**a**, **b**, **e**) and hypoglossal nerve (HG) (**c**, **d**) in normoxia and hypoxia in animals following vehicle (Sham), and 6-OHDA (6-OHDA) injection. Results are expressed as a percentage of the baseline nerve activity before hypoxia. All values are mean ± SEM. *N *normoxia, *R *recovery after the hypoxic stimulus. *^#^*p* < 0.05; **^##^*p* < 0.01, *—statistical significance in comparison to normoxic value expressed as 100% (*N*), #—statistical significance between corresponding points of hypoxic response present in 6-OHDA and Sham groups (*n* = 7–9 per group)

The frequency of nerve discharge was measured based on the activity of the phrenic nerve and it was the same as the frequency of the hypoglossal bursts. Raw values of nerve discharge frequency during normoxia did not differ between both analyzed groups; in 6-OHDA group it was 40 ± 4 and in sham rats 45 ± 4 bursts/min (*p* = 0.2). Baseline amplitude values of PHR activity were not significantly different between groups and in sham rats it was 1.16 ± 0.4 a.u., in 6-OHDA animals 1.43 ± 0.2 a.u. (*p* = 0.6). Figure 2 depicts changes in the phrenic nerve activity as percentage of control normoxic breathing. Frequency of phrenic nerve discharges increased during hypoxic stimulus and the increase was similar in both neurological states (Fig. 2e). Amplitude of the PHR in lesioned rats exhibited significantly higher values at 15 s (13%), 30 s (15%) and 45 s (12%) of response to hypoxia (Fig. 2a). PHR minute activity was augmented (26%) to a higher degree in 6-OHDA than in sham rats at first 15-s response to low oxygen (Fig. 2b).

Experiments in anesthetized rats on respiratory nerve activity showed no differences between both neurological states in time of hypoxia application to apnea appearance. 6-OHDA rats achieved apnea after 35 ± 3 s, sham rats after 29 ± 6 s of hypoxia exposure (*p* = 0.1). Time duration of apnea did not vary between groups, as well (*p* = 0.6). For 6-OHDA rats it was 42 ± 4 s versus sham 53 ± 15.

#### Activity of hypoglossal nerve in normoxia and in response to hypoxia in vehicle and 6-OHDA-treated rats

Baseline values of amplitude of HG nerve did not differ significantly between tested groups; in sham rats it was 0.66 ± 0.3 and in 6-ODA-lesioned 1.07 ± 0.3 a.u. (*p* = 0.3). Hypoglossal nerve activity expressed as a percentage of baseline normoxic activity displayed augmented amplitude and minute activity in both groups of animals during hypoxia (Fig. 2c, d). However, changes in amplitude of 6-OHDA-treated rats in response to hypoxia were substantially magnified (Fig. 2c). Significant growth of amplitude of PD model rats exceeded amplitude of sham ones over 55% at 15 s and 100% at 30 s of the reaction.

Minute activity: product of amplitude and frequency of nerve discharge was much more increased in 6-OHDA rats, as well (Fig. 2d). Significantly higher response to hypoxia over 90% than in the sham rats was observed at 15 s.

**Fig. 3:**
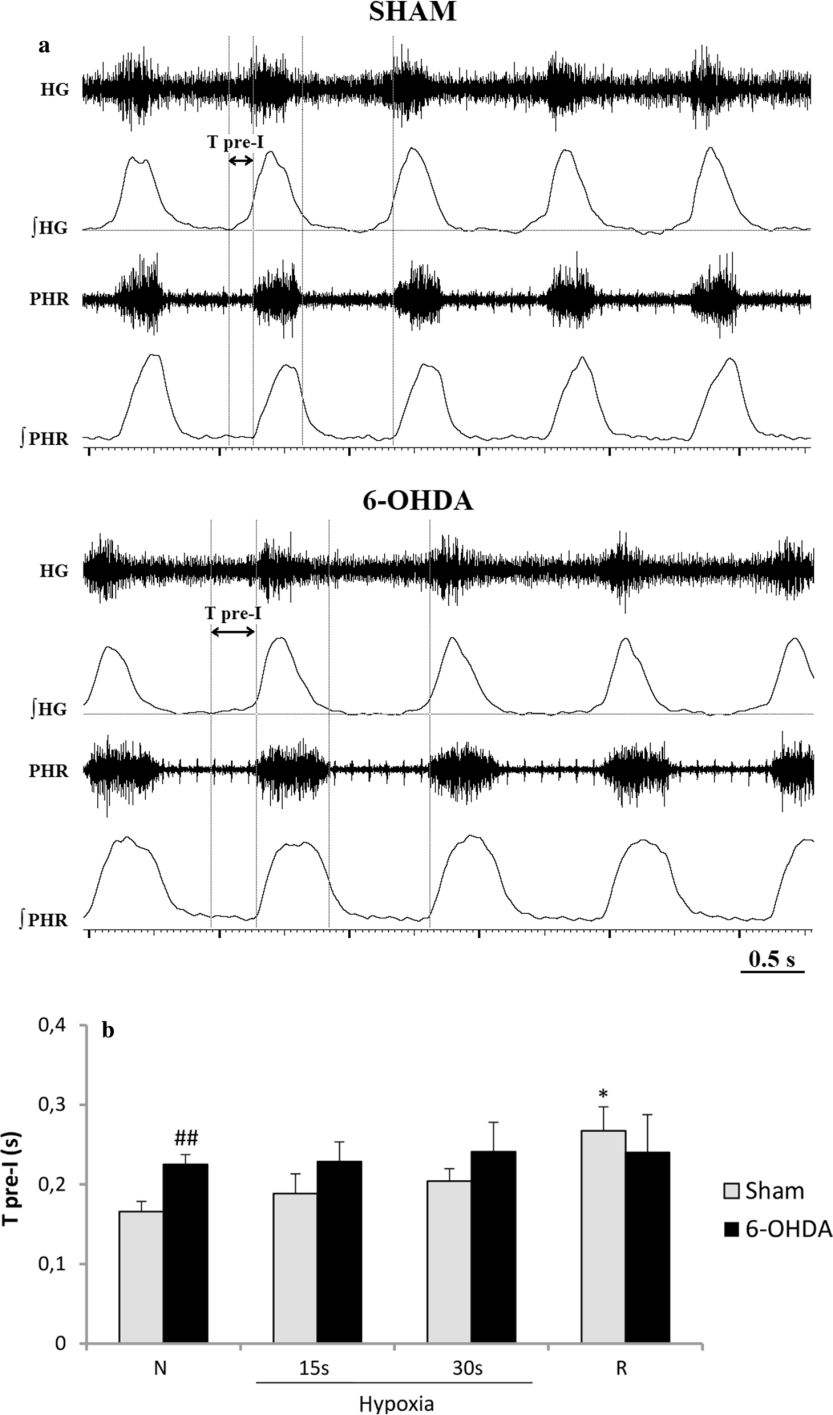
Sample recordings of PHR and HG nerve activity (raw and integrated signals—∫) during normoxia (**a**) and changes of pre-inspiratory time of HG nerve activity (T pre-I) in respiratory response to hypoxia (**b**) after injection of vehicle (Sham) or 6-OHDA into the third brain ventricle. Note visible more prolonged T pre-I in 6-OHDA-treated rats in normoxia. All values are given as mean ± SEM. *N* normoxia, *R* recovery after the hypoxic stimulus. *p < 0.05; ^##^*p* < 0.01. *—statistical significance versus the respective normoxia (N) value, #—significance between corresponding values in 6-OHDA and Sham group (*n* = 6–7)

Analysis of pre-inspiratory time of HG nerve activity (T pre-I) showed that it was much prolonged in 6-OHDA rats in normoxia (Fig. 3a, b). There were no differences in length of T pre-I between both groups during hypoxia and recovery time. Remaining analyzed parameters such as: Ti, Te, Tc, A pre-I were not statistically different between 6-OHDA and sham rats (data not shown).

**Fig. 4:**
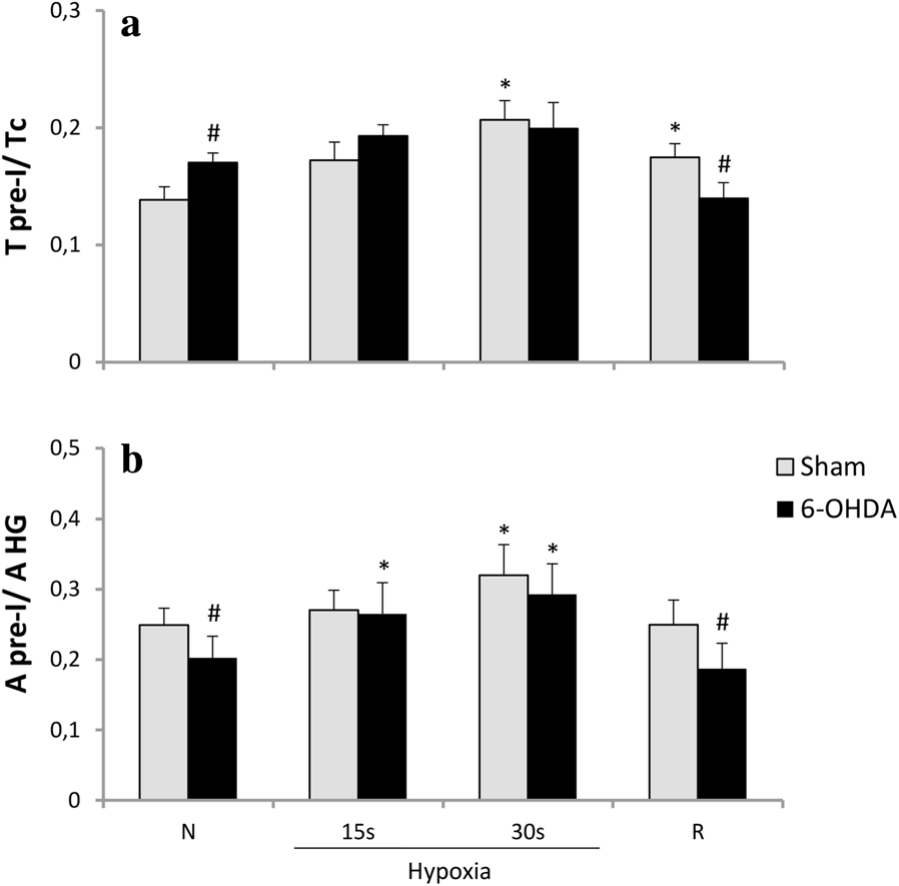
Changes in ratio of pre-inspiratory time of HG to a total length of the respiratory cycle (T pre-I/Tc) (**a**), and ratio of the pre-inspiratory hypoglossal nerve amplitude to the inspiratory HG peak amplitude (A pre-I/A HG) (**b**) in respiratory response to hypoxia after injection of vehicle (Sham) or 6-OHDA into the third brain ventricle. All values are given as mean ± SEM. *N* normoxia, *R* recovery after the hypoxic stimulus. *^#^*p* < 0.05. *—statistical significance versus normoxia (N) value, #—significance between corresponding values in 6-OHDA and Sham group (*n* = 6–7)

Analysis of ratio of pre-inspiratory time of HG nerve to the total length of the respiratory cycle (T pre-I/Tc) displayed significant changes between examined neural states during normoxia and recovery time (Fig. 4a). PD model rats had increased ratio during air breathing and decreased while recovery from hypoxia. Ratio of pre-inspiratory amplitude to the inspiratory peak amplitude of HG (A pre-I/A HG) in 6-OHDA rats was significantly decreased in normoxia (20%) and during post-hypoxic recovery time (28%) (Fig. 4b).

#### Ventilatory response after peripheral D_2_ receptors stimulation and blockade in vehicle and 6-OHDA-treated rats

**Fig. 5:**
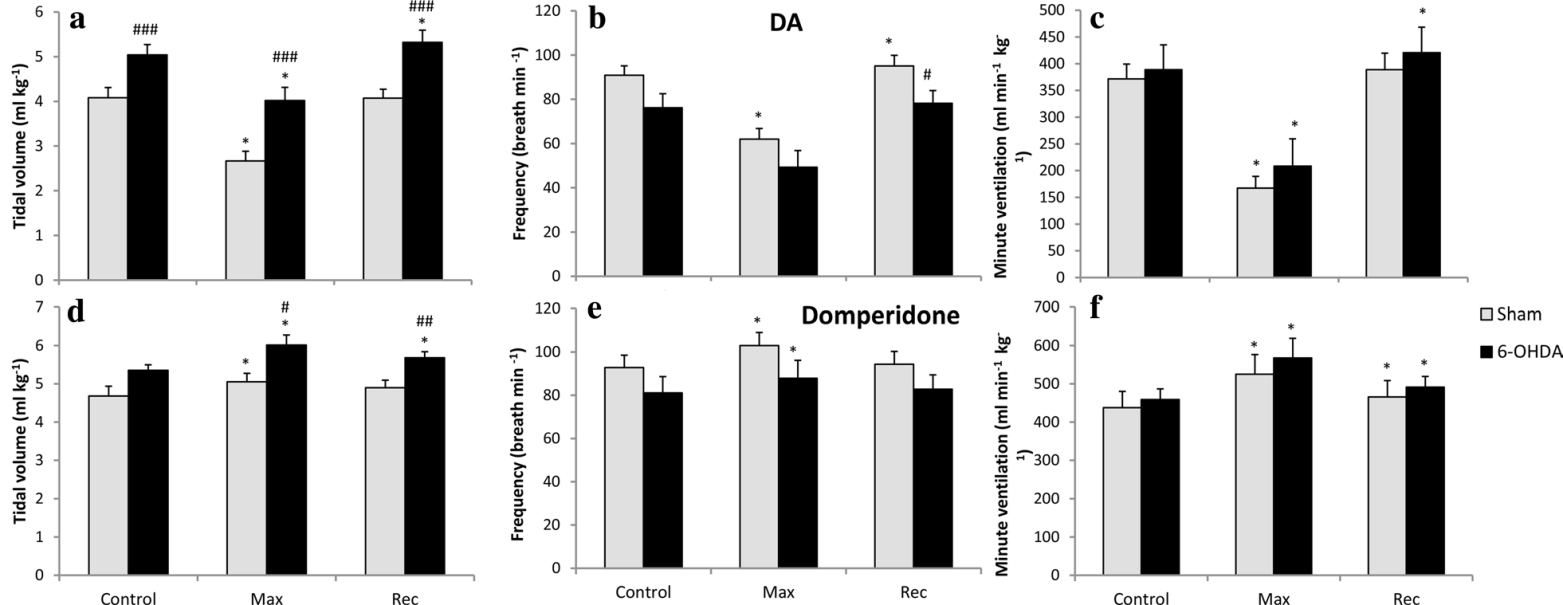
Effect of dopamine (**a**–**c**) and domperidone (**d**–**f**) i.v. injections on tidal volume, frequency of breathing and minute ventilation during normoxia in Sham rats and 14 days after ICV 6-OHDA treatment. Control—before treatment, Max—maximal respiratory changes after drug administration, *R* recovery after max. All values are mean ± SEM. *^#^*p* < 0.05; ^##^*p* < 0.01; ^###^*p* < 0.001. *—statistical significance versus baseline value before drug injection, #—statistical significance versus the corresponding Sham value (*n* = 6–7 per group)

In conscious 6-OHDA-treated rats normoxic respiratory parameters presented in Fig. 1 were changed in comparison to sham-operated animals. In similar way in this experiment performed under anesthesia tidal volume was significantly augmented in PD animals in normoxic breathing (Fig. 5a). Frequency of breathing decline observed after anesthesia in PD model rats did not exhibit significance like in plethysmography experiment, however the trend is similar (Fig. 5b). Opposing changes in V_T_ and f had no influence on changes in control minute ventilation (Fig. 5c).

Intravenous injection of dopamine induced prompt, short-living decrease of all respiratory parameters in neurotoxin and vehicle-treated rats (Fig. 5a–c). DA treatment significantly declined V_T_, f and V_E_ (to 46% of control values). The effect lasted 30 s.

Domperidone intravenous treatment caused increase in tidal volume and in frequency of breathing resulting in minute ventilation augmentation in sham (20%) and 6-OHDA (23%) rats (Fig. 5d–f). The effect lasted to 3 min after injection. PD model rats achieved more augmented V_T_ in point of maximal response and during recovery (Fig. 5e), but the change was the consequence of augmented tidal volume in 6-OHDA-treated rats at baseline values. Overall these data show that there is no difference between sham and PD animals in response to activation and blockade of peripheral D_2_ receptors.

### Body weight

Rats on the beginning of experiment before 6-OHDA lesion weighted 245 ± 2 g. Two weeks after neurotoxin injection body mass of animals increased to 259 ± 7 g. Sham group after vehicle injection presented increase in body weight from 256 ± 3 to 318 ± 10 g. Gain of body mass in both groups was significantly different (*p* < 0.01); in 6-OHDA rats it was 5% ± 3%, in sham rats 24% ± 4%.

### Behavioral open field test

**Fig. 6:**
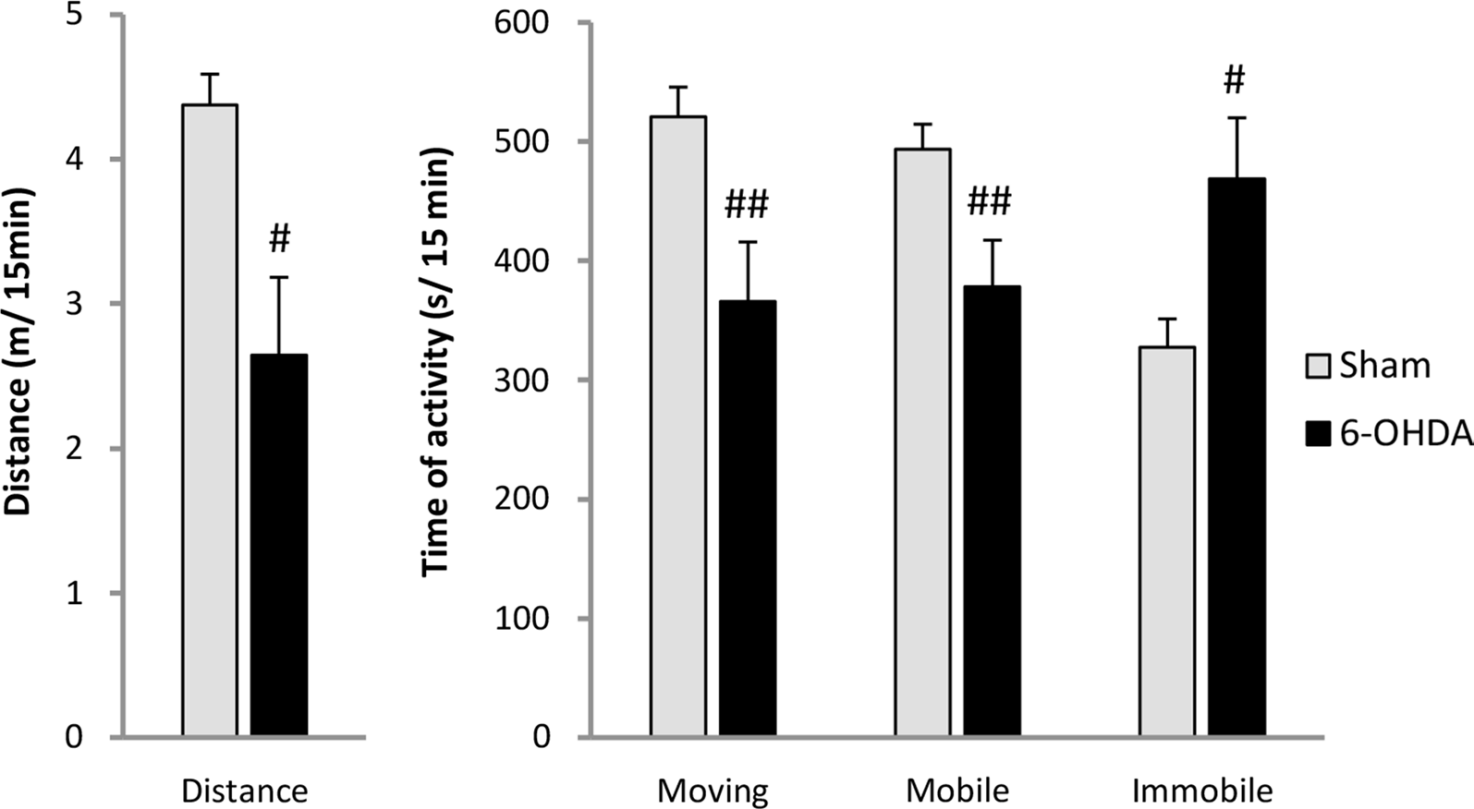
Changes of the motor activity during 15 min of behavioral open field test between vehicle (Sham) and 6-OHDA-treated rats. All values are given as mean ± SEM. ^#^*p* < 0.05; ^##^*p* < 0.01 significance versus Sham group (*n* = 7–9)

6-OHDA-injected rats showed decreased motor activity during 15 min of behavioral open filed test in comparison to vehicle-treated animals (Fig. 6). PD model rats presented reduced total distance length (33%) and time of moving (30%) and prolonged the time of immobility (43%) in comparison to sham animals.

### Content of monoamines and metabolites in the striatum and brainstem

**Fig. 7:**
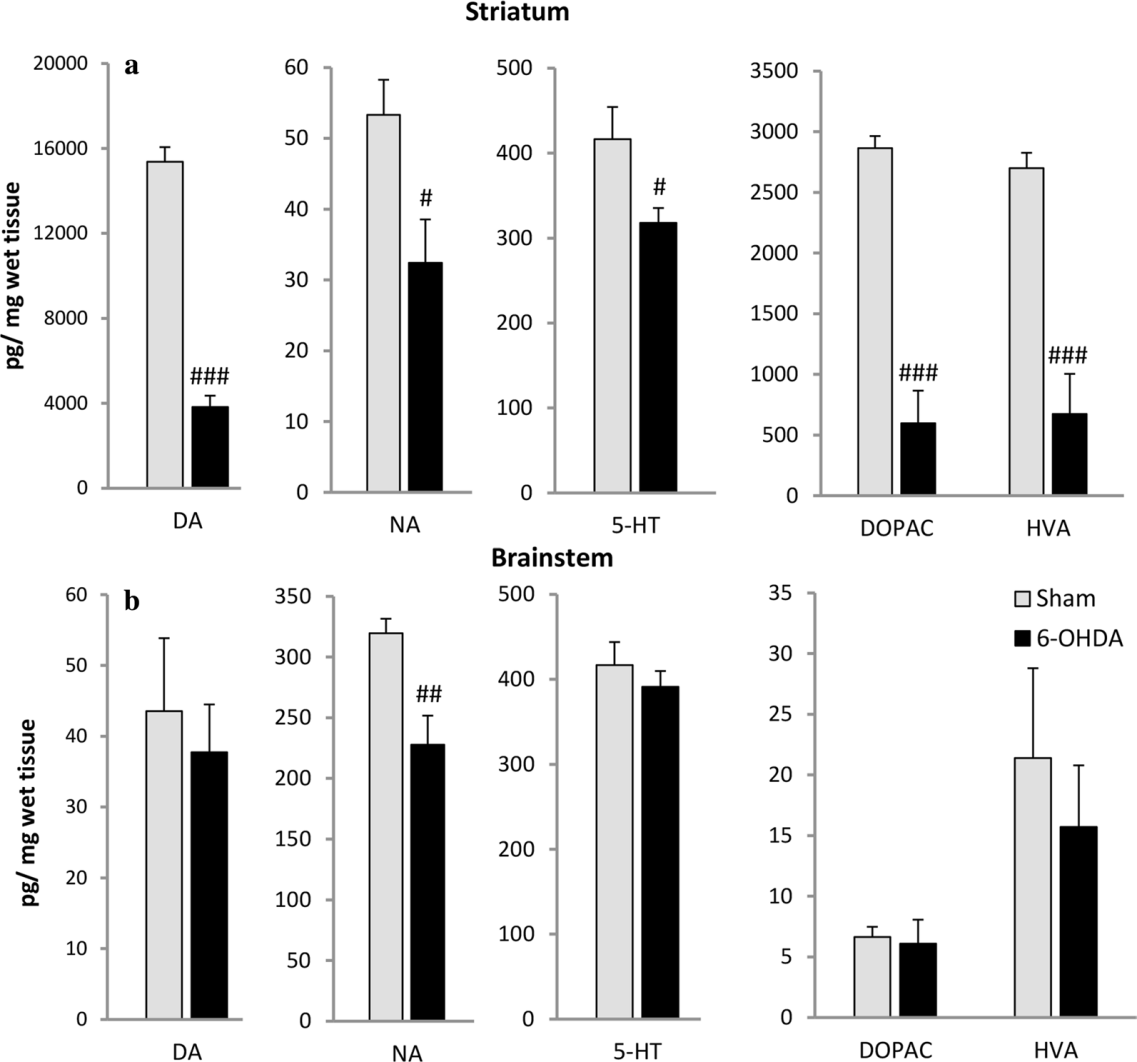
Mean concentration of dopamine (DA), noradrenaline (NA), serotonin (5-HT), 3,4-dihydroxyphenylacetic acid (DOPAC) and homovanillic acid (HVA) in the striatum (**a**) and brainstem (**b**) of Sham and 6-OHDA rats, evaluated by HPLC detection ex vivo*.* The data are expressed as mean ± SEM. ^*###*^*p* < 0.001, ^##^*p* < 0.01, ^#^*p* < 0.05 significance versus Sham group (*n* = 7–8)

ICV injections of 6-OHDA resulted in 75% reduction of striatal DA concentration compared to the sham-operated rats accompanied with diminished metabolites: DOPAC level by 80% and HVA by 75%. Striatal NA and 5-HT were also reduced in the 6-OHDA-treated group by 40% and 24%, respectively (Fig. 7). In the brainstem only NA was significantly decreased by 28% in comparison to sham rats (Fig. 7). The content of 5-HIAA, metabolite of 5-HT was not significantly different between examined groups (data not shown). The level of MHPG, NA metabolite was undetectable.

## Discussion

Our study demonstrated that substantial depletion of DA in the striatum resulted in normoxic respiratory pattern changes characterized by slowing breathing and augmented tidal volume and altered activity of phrenic and hypoglossal nerve. The latter was investigated for the first time in bilateral PD model produced with ICV 6-OHDA injection. We also managed to confirm the lack of lesion effect on peripheral D_2_ receptor functioning.

Basic difference observed in the bilateral model in comparison to our previous study, when we applied 6-OHDA to one hemisphere [11, 26, 27], is altered to higher extent hypoglossal nerve activity and for the first time also augmented phrenic nerve amplitude during hypoxia. The pre-inspiratory time and the ratio of T pre-I/Tc were increased during baseline breathing, which was parallel to diminished ratio of pre-inspiratory HG nerve amplitude to the inspiratory HG peak amplitude. Hypoglossal pre-inspiratory activity is regarded as responsible for the patency of the pharyngeal airway before inspiration [28–30]. Hypoglossal nerve facilitates opening of the glottis and prevents collapse of the upper airways, which is the main reason of apnea [31]. Given these facts, altered ratios may indicate disturbances in maintenance of patency of the upper airway respiratory tract during the pre-inspiratory phase.

The analysis of the phrenic and the hypoglossal nerve activities in the bilateral 6-OHDA model demonstrated augmented response to hypoxia in amplitude and minute activity of both nerves. The increased amplitude of PHR activity during hypoxia was present only in the current model in contrast to previously studied unilateral ones [7, 26]. In these models the intact hemisphere plausibly compensated the depletion of DA and responded for the unaltered phrenic nerve activity.

Dopamine playing a crucial role in the oxygen detection in the peripheral chemoreceptors of the carotid body [32], in the CNS is regarded to be involved in the depressive phase of the respiratory response to hypoxia [16, 17]. Exogenously applied DA was reported to inhibit the activity of the hypoglossal and phrenic nerves [33], thus we suppose augmented amplitudes of both nerves can result from 6-OHDA-evoked DA depletion. In correspondence with our results, striatal DA loss evoked with this neurotoxin produced impairment of motoric tongue functions [34, 35], which indicated changes in control of tongue, innervated with the HG nerve. What is interesting, changed expression of D_1_ and D_2_ receptors have been demonstrated within the nucleus of the hypoglossal nerve (HG) of 6-OHDA-lesioned rats [36].

We found that stimulation or blockade of peripheral DA receptors produced similar respiratory responses in both sham and PD model rats, which means that carotid body DA receptors remained unaffected by central 6-OHDA injection. This is in contrast to Białkowska et al. [9] study, where lack of domperidone stimulatory effect on respiration was reported in reserpine rat model of parkinsonism. In this case, however reserpine depletes dopamine and other monoamines in the brain and in the periphery [37], thus most probably also in the carotid body. Dopamine respiratory effect has not been tested in PD model rats so far, and when applied i.v. it produced short-lived depression of ventilation as it was confirmed in sham group and earlier study in healthy cats [38]. The latter study showed also that carotid body denervation eliminated DA-induced respiratory depression, which confirmed that receptors located in these structures were involved.

Other neurotransmitter systems like serotonergic or noradrenergic have been postulated to contribute to respiratory PD abnormalities [8, 12, 39–41].

Previously in a unilateral model (medial forebrain injection), we demonstrated that 6-OHDA treatment depleted not only DA, but also decreased the content of 5-HT in the injured striatum and in both sides of the brainstem, with no effect on NA concentration [27]. ICV lesion reduced DA with lower impact on both NA and 5-HT levels in the striatum. The only change in the brainstem was significantly decreased NA regardless of desipramine pretreatment. Thus, it seems that depending on the site of 6-OHDA injection disturbance in various monoaminergic systems can be obtained. What is more important there is growing evidence that noradrenergic neurons of the brainstem locus coeruleus (LC) also degenerate in PD and can be responsible for motor and non-motor deficits in the course of disease [39]. The catecholaminergic terminals in the brainstem project to regions involved in respiratory control [42, 43] and NA provides a powerful stimulus for breathing in normoxia, hypoxia and hypercapnia [44–47]. What is more, α_1_-adrenergic receptors have been shown to be the main postsynaptic mediator of adrenergic excitation in HG motoneurons [48]. There is also evidence that activation of α_1_-adrenergic located near the phrenic motor nucleus elicits phrenic long-term facilitation [49].

We can speculate that NA reduced by 28% in the brainstem could be responsible at least is some part for respiratory changes present after 6-OHDA. Substantial lesion of noradrenergic neurons of LC [46] and most of the bulbospinal catecholaminergic neurons (C1 and A5 group) resulted in blunted ventilatory response to hypoxia [50]. We observed rather tendency to increased hypoxic minute ventilation, thus this moderate depletion of NA present in our study seems to be compensated. Intriguing fact is that 6-OHDA lesion of dopaminergic neurons evokes overactivation of LC noradrenergic neurons suggesting their impact could be augmented in this model [51]. It is plausible that in the present study 6-OHDA injection produced brainstem noradrenergic neurons loss, evidenced by declined concentration of NA, which in turn led to the overactivity of remaining noradrenergic neurons. This compensatory mechanism for the dopaminergic dysfunction in PD may be responsible for slight minute ventilation change with tendency to nonsignificant increase despite the increase of tidal volume and reduction of respiratory rate during air breathing and hypoxia. The same could be observed with augmented amplitudes of hypoglossal and phrenic nerves.

After striatal bilateral lesion, LC was not affected and the main observed respiratory effect was the decrease of frequency of breathing, without any changes in tidal volume [8]. Yet in our study ICV neurotoxin application was apparently able to affect brainstem noradrenergic system what was reflected in diminished NA concentration. Therefore, the normoxic tidal volume increase reported by us could be the effect of aforementioned 6-OHDA-induced overactivity of noradrenergic neurons [51]. The role of NA system in breathing modulation was confirmed by Oliveira et al. [10] in conclusion that spared NA neurons of LC could be responsible for maintaining chemoreflex sensitivity, which was reduced in response to CO_2_ in 6-OHDA-lesioned rats with additionally ablated noradrenergic LC neurons.

Respiratory alteration observed in the present study coexisted with behavioral changes, confirmed by the open field test estimating locomotor activity and motivation of animals to explore the surrounding environment. Significantly longer immobility time and shorter distance travelled by ICV 6-OHDA rats corresponded to results obtained after bilateral partial intranigral lesion [52, 53]. It is well known that 6-OHDA-treated rats revealed an increase in immobility time which is typical for anhedonia (i.e., decreased motivation/responsiveness to reward) [54]. We cannot exclude that immobility/anhedonia was partially mediated through the alterations in the mesolimbic dopamine system [55]. We did not examine the latter, but the study by Rodriguez-Rodríguez-Diaz et al. [56] reported that ICV 6-OHDA injection was effective in producing loss of dopaminergic neurons in the ventral tegmental area.

## Conclusion

In conclusion, we suggest that respiratory changes appearing in ICV 6-OHDA model of PD results from the changed control of breathing at the brainstem level related to impairment in two neurochemical systems: substantial striatal depletion of DA and significant depletion of the brainstem NA. Magnified amplitudes of HG and PHR nerve activity during hypoxia and the changes in pre-inspiratory activity of HG nerve are more pronounced in the bilateral 6-OHDA model. Yet, we suggest cautious selection of 6-OHDA model to studying non-motor symptoms since depending on the site of the neurotoxin injection specific deficits beyond the dopaminergic systems can be achieved.

## Data Availability

All data generated or analyzed during this study are included in this published article.

## Abbreviations

AHG: Amplitude of hypoglossal nerve activity
A pre-I HG: Amplitude of the pre-inspiratory hypoglossal nerve activity
5-HIAA: 5-Hydroxyindolacetic acid
5-HT: Serotonin
6-OHDA: 6-Hydroxydopamine
CNS: Central nervous system
DA: Dopamine
DOPAC: 3,4-Dihydroxyphenylacetic acid
f: Frequency of breathing
HG: Hypoglossal nerve
HPLC: High-performance liquid chromatography
HVA: Homovanillic acid
ICV: Intracerebroventricular injection into III ventricle
LC: Locus coeruleus
MFB: Medial forebrain bundle
MHPG: 3-Methoxy-4-hydroxyphenylglycol acid
NA: Noradrenaline
NTS: Solitary tract nucleus
PD: Parkinson’s disease
PHR: Phrenic nerve
Te: Expiratory time
Ti: Inspiratory time
Tc: Total respiratory cycle time
T pre-I HG: Pre-inspiratory hypoglossal activity
V_E_: Minute ventilation
V_T_: Tidal volume
